# Development and psychometric evaluation of a new domain‐specific coparenting measure: Coparenting Children's Emotion Scale

**DOI:** 10.1111/famp.13031

**Published:** 2024-08-21

**Authors:** Christina C. Ambrosi, Phillip S. Kavanagh, Subhadra Evans, Sophie S. Havighurst

**Affiliations:** ^1^ Justice and Society University of South Australia Magill South Australia Australia; ^2^ Mindful: Centre for Training and Research in Developmental Health, Department of Psychiatry University of Melbourne Melbourne Victoria Australia; ^3^ Discipline of Psychology University of Canberra Bruce Australian Capital Territory Australia; ^4^ Centre for Social and Early Emotional Development Deakin University Burwood Victoria Australia

**Keywords:** cooperative coparenting, coparenting measure, coparenting relationship, emotion, emotion socialization, supportive coparenting, undermining coparenting

## Abstract

Caregivers play an integral role in supporting children's development, not only through their individual parenting practices but also how they work together as coparents. The literature on coparenting is extensive; however, most of the research has relied on global measures to assess the quality and functioning of the coparenting relationship. Examining the coparenting relationship with domain‐specific measures enables a deeper understanding of this complex family process. One domain of particular interest is emotion socialization given the vast and long‐term consequences emotion socialization has on children's emotional, social, behavioral, and psychological functioning. Emotion socialization literature would benefit from a domain‐specific coparenting measure, as researchers have rarely explored how coparents work together when responding to their children's emotions (i.e., coparenting children's emotions). As such, an emotion‐focused coparenting measure could address gaps in both coparenting and emotion socialization literature. This study outlines the development and psychometric evaluation of a domain‐specific measure of coparenting, the Coparenting Children's Emotion Scale (CCES), which assesses how parents work together when responding to their children's emotions. In the current study, the factor structure, reliability, and validity of the CCES were examined in an Australian sample. Findings from exploratory and confirmatory factor analyses showed that the CCES comprises two subscales that capture coparents' levels of support/cooperation and undermining. In the current sample, both CCES subscales demonstrated good to excellent internal consistency, and good convergent and concurrent validity. The CCES will provide researchers and practitioners with a domain‐specific measure to use in exploratory and intervention research.

In recent years, there has been an increased focus on understanding coparenting relationships (e.g., Eira Nunes et al., 2021; Pruett et al., 2017). The coparenting relationship refers to the way parents/caregivers work together to raise a child (McHale et al., 2019). Coparenting is conceptualized as a triangular family process, involving a child and at least two adults sharing responsibility for the care and upbringing of that child (McHale et al., 2019). As all coparenting interactions are centered around raising a child (McHale & Irace, 2011), the coparenting relationship exists irrespective of the romantic relationship between parents (e.g., divorce and separation). As such, the coparenting relationship and romantic relationship are related yet distinct subsystems (Maršanić & Kušmić, 2013), with the romantic relationship typically being dyadic (i.e., two adults), involving different stages of love (Bode & Kushnick, 2021) and ongoing commitment and satisfaction (Campbell & Fletcher, 2015). Each coparenting relationship differs in the degree of support, cooperation, unity, conflict, and undermining between parents (McHale et al., 2019).

The coparenting relationship plays a pivotal role in child, parent, and family functioning. A meta‐analysis by Teubert and Pinquart (2010) revealed that higher levels of coparenting cooperation and agreement, and lower levels of conflict between parents were associated with improved child social functioning, and reduced internalizing and externalizing symptoms. The coparenting relationship shapes children's adjustment by influencing the family's emotional stability (Davies & Cummings, 1994), parenting practices (Bonds & Gondoli, 2007; Margolin et al., 2001; Morrill et al., 2010), parental emotional availability (Sturge‐Apple et al., 2006), and parent–child relationships (Feinberg & Kan, 2008; Teubert & Pinquart, 2010). Research has also shown that the quality of the coparenting relationship is related to interparental communication, parenting stress, and romantic relationship quality. Findings by Shimkowski and Schrodt (2012) show that coparent supportive and antagonistic communication were moderately to strongly associated with interparental demand/withdrawal patterns. More recently, Zemp et al. (2020) reported a moderate correlation between constructive interpersonal conflict and coparenting. Coparent relationship quality is also shown to be strongly associated with ineffective arguing and couple conflict (Feinberg et al., 2012). Furthermore, research indicates that mothers and fathers both report small to moderate correlations between supportive and undermining coparenting and parental stress (e.g., Solmeyer & Feinberg, 2011). The extant evidence‐base demonstrates that the coparenting relationship is central to family and child functioning; however, most studies have used global measures of coparenting to assess how parents work together across multiple domains of parenting. In comparison to global measures, domain‐specific measures can provide a tailored understanding of coparenting dynamics within a certain parenting domain (e.g., emotion socialization, education, or feeding), which can consequently inform targeted interventions.

## The history of conceptualising and measuring coparenting

Self‐report measures of coparenting have developed over time to reflect advances in the theoretical literature (see Molla Cusi et al., 2020 for a review). Throughout the 1980s, coparenting was viewed as a process occurring in post‐divorce families and was therefore solely assessed in separated parents (e.g., Ahrons, 1981). By the mid‐1990s, researchers expanded the coparenting concept to two‐parent, intact families (e.g., McHale, 1995, 1997). Measures such as the Family Experience Questionnaire (Frank et al., 1988), Parenting Alliance Inventory (Abidin & Brunner, 1995), Coparenting Scale (McHale, 1997), Coparenting Questionnaire (Margolin et al., 2001), and Perceptions of Coparenting Partners Questionnaire (Stright & Bales, 2003) were developed to provide measures of coparenting in married/de facto parent dyads. These measures assess various aspects of the coparenting relationship, including support, endorsement and respect, cooperation, sharing responsibilities, conflict, and triangulation.

In 2003, Feinberg unified research on divorced and intact families by developing a conceptual framework of coparenting. The framework highlights the dynamic nature of the coparenting relationship, outlining four overlapping domains: coparent support/undermining, childrearing agreement, division of labur, and joint management of family dynamics (Feinberg, 2003). To measure the multidimensional nature of coparenting, Feinberg et al. (2012) developed the Coparenting Relationship Scale (CRS). The CRS comprises items from previous measures of the parenting alliance and coparenting (Abidin & Brunner, 1995; Cordova, 2000; Frank et al., 1988; Margolin, 1992; McHale, 1997), as well as new items created by Feinberg et al. (2012). The CRS provides researchers with a global measure of coparenting relationship quality, which has been used across diverse samples in exploratory research (e.g., Riina & Feinberg, 2018) and to evaluate coparenting interventions (e.g., Abbass‐Dick et al., 2015). The CRS has also been used internationally and translated into different languages (Portuguese—Carvalho et al., 2018; French—Favez et al., 2021; Swedish—Lee et al., 2020). Additional coparenting measures have been written in languages other than English including the Questionnaire on Perceived Support from the Former Partner (CARE) in Spanish (Yárnoz‐Yaben, 2010) and Coparenting Inventory for Parents and Adolescents in German (Teubert & Pinquart, 2011).

Global measures of coparenting are readily available (e.g., see Feinberg et al., 2012; Margolin et al., 2001); however, assessing how coparents navigate specific parenting domains and responsibilities can provide more nuanced insights into coparenting dynamics. Recent recognition of the need for domain‐specific coparenting measures led to the development of the Feeding Coparenting Scale (Tan et al., 2019), which assesses how parents work together to feed their children. The Feeding Coparenting Scale has been used to examine the relationship between feeding coparenting, food parenting practices, and child eating behaviors (e.g., Jansen et al., 2022; Sherrard & Tan, 2022), thus informing future childhood obesity prevention and interventions. Based on recent research by Douglas et al. (2024), it appears general coparenting quality does not capture the intricacies of how coparents interact when feeding their children. Accordingly, global measures of coparenting may lack necessary context specificity for researchers and practitioners who are developing and evaluating targeted parenting interventions.

## A domain‐specific measure: Coparenting children's emotion

A domain‐specific measure should be developed to explore coparenting in the context of emotion socialization (i.e., how parents express, discussion, and respond to children's emotions). It is beneficial to understand the way coparents work together to navigate emotion socialization, given the impact parental emotion socialization has on children's emotional competence (i.e., ability to expression, understand, and manage emotion), and consequently their social relationships, behavioral adjustment, mental health, and educational achievement (e.g., Denham, 2019; Jones et al., 2015). A domain‐specific coparenting measure could specifically assess how parents work together when responding to their children's emotions (i.e., coparenting children's emotion).

Research into parent emotion socialization has expanded rapidly over the last 20 years; however, the existing evidence‐base mostly focuses on mothers, with minimal research published on fathers and how parents work together when addressing children's emotions (Eisenberg, 2020). Despite the gaps in emotion socialization literature, research has highlighted the role that family relationships play in children's emotional development (e.g., Cowan & Cowan, 2005; Morris et al., 2007). From a family systems perspective, the family unit is an organized whole consisting of interconnected subsystems (e.g., coparent relationship, parent–child relationship; McHale & Sullivan, 2008; Minuchin, 1974); therefore, the functioning of one subsystem naturally infiltrates through the family (McHale & Sullivan, 2008). The spill‐over hypothesis suggests that difficulties between coparents have implications for parents' interactions with their children (Davies et al., 2009; Sturge‐Apple et al., 2006), such that parents in more supportive coparenting relationships are typically more sensitive to their children's needs (Zemp et al., 2018). Coparenting adults who are willing to communicate, collaborate, and support one another are better able to jointly attune to their children's emotional needs (McHale et al., 2019). In contrast, parents in more undermining coparent relationships have increased emotional unavailability and engage in less responsive parenting, which consequently increases the likelihood of child maladjustment (Sturge‐Apple et al., 2006; Zemp et al., 2018). It is pertinent to explore how parents work together when responding to their children's emotions, as coparenting uniquely contributes to child's development beyond the impact of individual parenting practices (Bonds & Gondoli, 2007; Margolin et al., 2001). As such, it is plausible that coparenting children's emotions uniquely influences children's emotional competence beyond each parents' individual emotion socialization practices.

Emotion socialization research, predominantly conducted with heterosexual dyads, consistently shows that mothers and fathers report differences in their emotion socialization practices. Mothers typically provide more supportive responses to their children's emotions (e.g., sadness and fear) compared to fathers (e.g., Cassano et al., 2007). Additionally, compared to mothers, fathers are typically less likely to discuss emotions with their children (Zaman & Fivush, 2013) and are more likely to be critical and dismissive when children express emotions (Cassano et al., 2007; Engle & McElwain, 2010; Gottman et al., 1996; Nelson et al., 2009; Wong et al., 2009). Differences in parents' emotion socialization practices are not inherently damaging for children, rather initial research shows variation in parents' emotional responding can be beneficial for children's emotional understanding (e.g., McElwain et al., 2007). However, it is helpful to understand the manner in which parents navigate their potential differences. For example, coparents who are highly undermining may be extremely critical of how the other parent responds to their children's emotions, whereas other coparents who are supportive may engage in collaborative discussions about their differences. Accordingly, a coparenting children's emotion measure does not aim to quantify differences between parents' emotion socialization practices, but instead assess the way parents do or do not work together to respond to their children's emotions.

A new measure of coparenting children's emotions could be used by researchers and practitioners. As demonstrated in research conducted with the Feeding Coparenting Scale (e.g., Jansen et al., 2022; Sherrard & Tan, 2022), a new domain‐specific measure focused on emotion socialization could provide researchers with the ability to explore connections between coparenting children's emotions, parents' emotion socialization practices, and children's emotional competence. For example, researchers may examine the extent to which undermining in coparenting children's emotion is positively correlated with emotion unavailability and emotion dismissive/disapproving practices (e.g., minimizing or criticizing children's emotions), and whether support/cooperation in coparenting children's emotion is positively correlated with emotional sensitivity and emotion coaching (e.g., noticing and validating children's emotions). Developing an understanding of the role of coparenting children's emotions can inform intervention development and evaluation.

A coparenting children's emotion measure would be most appropriate and relevant for emotion‐focused parenting programs, which are interventions underpinned by emotion socialization theory and aim to improve children's emotional competence through various mechanisms of change (Havighurst et al., 2020). Emotion‐focused parenting programs that promote children's emotional competence by enhancing parents' emotional socialization practices and/or improving the coparenting relationship could use a coparenting children's emotion measure to assess intervention outcomes. It is plausible that coparenting children's emotions also has a moderating effect, such that it influences the extent to which parents use newly learnt emotion socialization practices at home (i.e., skill enactment). That is, supportiveness and undermining in coparenting children's emotions may act as enablers and/or barriers to change within the family system. Parents with higher supportiveness in coparenting children's emotions may achieve greater program benefits, whereas parents with higher levels of undermining in coparenting children's emotions may have greater difficulty implementing new strategies. By understanding the moderating effects of coparenting children's emotions, researchers and practitioners can develop and refine emotion‐focused parenting programs and make recommendations about which families will benefit most from attending such programs.

## Modifying the coparent relationship scale

The CRS (Feinberg et al., 2012) can be modified to create a domain‐specific measure of coparenting children's emotions as it captures the multi‐dimensional nature of the coparent relationship and has demonstrated good psychometric properties in diverse samples. The CRS demonstrated adequate to excellent reliability in samples from various countries, including the United States (e.g., Feinberg et al., 2012; Parent et al., 2016), Canada (Abbass‐Dick et al., 2015), Portugal (e.g., Lamela et al., 2018; Pinto et al., 2019), and Italy (Giannotti et al., 2021). In previous research the CRS has also demonstrated good internal consistency in samples of caregivers in different stages of parenthood (e.g., prenatal, parents of infants through to adolescents). Additionally, the CRS demonstrated good convergent validity in a sample of heterosexual coparenting couples in the United Sates of America, with moderate to strong associations reported between the CRS and theoretically related constructs, such as quality of the marital relationship, couple love, couple conflict, ineffective arguing, and divorce proneness (Feinberg et al., 2012). A study with Portuguese mothers also provided evidence of satisfactory convergent, construct, and discriminant validity (Lamela et al., 2018). Furthermore, the measure has previously been adapted to specific populations (e.g., pre‐natal fathers; Pinto et al., 2019) and into a daily measure of coparenting (Daily Coparenting Scale; McDaniel et al., 2017). Given the psychometric properties demonstrated by the CRS, the measure provides a framework that can be modified to focus on coparenting children's emotions.

## The current study

The current study outlines the development of the Coparenting Children's Emotion Scale (CCES) and aims to evaluate the psychometric properties of the measure by examining the factor structure, reliability, and validity in a sample of Australian coparents. Convergent validity was assessed by examining correlations between the CCES and CRS. Concurrent validity was assessed by examining correlations between the CCES and constructs theoretically and empirically related to coparenting: interparental communication, parenting stress, and romantic relationship quality.

## METHOD

### Scale development

The development of the CCES started with the first author (CA) reviewing items from the CRS (Feinberg et al., 2012). CRS items were modified to specifically assess how parents work together when focused on their children's emotions. For example, the CRS item “*We often discuss the best way to meet our child's needs*” was modified to “*We often discuss the best way to meet our child's emotional needs*” in the CCES. CRS items were also modified to be inclusive of all coparenting dyads (e.g., married/de facto, separated/divorced) to ensure the measure is not limited to coparents who are romantically involved. For example, the sentence stem “*my partner and I*” was changed to “*the other parent and I*”. Five additional items were adapted from Stright and Bales (2003) and CA created six additional items (e.g., “*In general, I think we work well together to support our child's emotional wellbeing*”; “*The other parent and I have arguments about the best way to respond to our child's emotions*”). CA generated the modified items, which were then reviewed by the co‐authors. A total of 46 items were retained for data collection and analysis. See Supplementary Materials for a full list of items.

### Participants and procedure

Three hundred eighty parents/caregivers^1^ (51.3% female, 13.7% male, 35% did not report) were recruited to complete an online survey hosted on SurveyMonkey, containing the newly developed CCES alongside other measures. Two hundred seventy‐one parents/caregivers completed the survey. The survey was advertised via social media, with flyers also distributed at Australian schools, childcare centers, kindergartens, libraries, community centers, Scout clubs, shopping centers, and via letterbox drops. Human research ethics approval was obtained from the University of South Australia (200494) and Catholic Education South Australia (201804). Individuals self‐selected to participate and provided informed consent online. Before commencing the survey, participants generated a unique code that could be used by both members of the coparenting dyad. Participating coparents were asked to share this code with one another to assist with recruitment, and therefore it was hoped that majority of the sample would be coparenting dyads. The codes were used to link participants' responses and separate coparents during data analysis to ensure the assumption of independence was not violated (i.e., data remained dependent in analyses). After entering the code, participants provided demographic information and completed measures of coparenting, interparental communication, and parenting stress. Participants who reported being in an intimate/romantic relationship (e.g., married, engaged, and dating) with the other parent/caregiver completed an additional measure relating to their current partner and romantic relationship. At the completion of the study, participants were asked to forward the survey link and their unique code onto the other parent/caregiver. Participants who completed the survey were given the opportunity to enter the draw for one of three $100 e‐gift cards. To increase recruitment, parents were offered an electronic parenting information pack after completing the survey.

The final sample consisted of 271 parents, of which 201 participants were sole respondents from their respective coparent dyads and 35 were coparenting dyads (i.e., both parents in the coparenting relationship; *n* = 70 parents). Participants' age ranged from 21 to 68 years (*M*_age_ = 38.86, *SD*_age_ = 8.08). Most participants identified as female (69.4%; 18.5% male; 12.1% did not specify). Most of the coparents were married/de facto (86.72%) and living together (86.30%). All parents lived in Australia, with a large proportion living in South Australia (66.79%). Most parents completed a university degree (57.96%) as their highest level of education, which is a similar percentage to Australian adults aged 35–44 years whose highest level of education is a university degree (58.3%; Australian Bureau of Statistics, 2021). The majority of parents reported gross household income above the Australian median household income ($88,452; Australian Bureau of Statistics, 2018). Most parents were raising one or two children under the age of 18 years (85.30%). The mean age of parents' first child was 7.95 years old (*SD* = 5.29, range = 18) and second child was 6.47 years old (*SD* = 4.65, range = 16). Parents were able to select up to three responses when asked to report their cultural/ethnic identity, and the majority (75.6%) of participants identified as Australian. See Table 1 for demographic information.

**TABLE 1:** Participant demographics.

Demographic variable	*n* (%)
Relationship status with the other parent/caregiver	
Married/de facto	235 (86.7%)
Divorced/separated	19 (7.0%)
Engaged	6 (2.2%)
Self‐identified as “coparents/partners in the family way”	3 (1.1%)
Other^a^	8 (3.0%)
Living arrangements	
Living together full‐time	234 (86.3%)
Living together part‐time (e.g., several days per week)	8 (3%)
Not living together	29 (10.7%)
Number of children	
One	108 (39.9%)
Two	123 (45.4%)
Three	32 (11.8%)
Four	6 (2.2%)
Six or more	2 (0.7%)
Location	
South Australia	181 (66.8%)
Other Australian states	46 (17.0%)
Did not specify	44 (16.24%)
Family total gross yearly household income	
<$60,000	41 (15.10%)
$60,000–$99,999	52 (19.2%)
$100,000–119,999	36 (13.3%)
$120,000–149,999	39 (14.4%)
$150,000 +	65 (24%)
Did not respond	38 (14%)
Highest level of education	
Less than high school/high school completion	27 (9.96%)
Certificate	18 (6.6%)
Apprenticeship/advanced diploma/diploma	36 (13.3%)
Bachelor's degree	62 (22.9%)
Post graduate degree/graduate diploma/graduate certificate	95 (35.1%)
Did not specify	33 (12.2%)
Cultural/ethnic identity	
Australian	205 (75.65%)
English	20 (7.38%)
Italian	15 (5.54%)
British	10 (3.96%)
Chinese	10 (3.96%)
Indian	10 (3.96%)
Scottish	7 (2.58%)
German	4 (1.48%)
Irish	4 (1.48%)
Australian Aboriginal	3 (1.11%)
Arab	3 (1.11%)
Maltese	3 (1.11%)
Russian	3 (1.11%)
Vietnamese	3 (1.11%)
Other^b^	

^a^ One participant (0.4%) reported never being married to the coparent; one participant (0.4%) reported having court‐mandated intervention order with the other coparent; two participants (0.8%) reported coparenting with a friend; two participants (0.8%) reported coparenting with a family member; two participants (0.8%) reported coparenting with someone they are dating seriously.

^b^ Cultural/ethnic identities that comprise <1% of the sample are not presented (e.g., Zimbabwean, Polish, Ukrainian, Greek, Colombian, Chilean, Macedonian, and Malay).

### Measures

#### Coparent relationship

The CRS (Feinberg et al., 2012) was used to provide a comparison between the CCES and a widely used measure of coparenting. The CRS is a 35 item, self‐report measure that assesses the quality of the coparenting relationship (Feinberg et al., 2012). All items were rated on a 7‐point scale (1 = *not at all true of us*; 7 = *very true of us*), except for exposure to conflict items which had different descriptive anchors (0 = *never*; 6 = *very often*). The wording of items was adjusted to ensure inclusivity (e.g., “partner” was changed to “the other parent/caregiver”). Items were averaged to create a global index score of coparenting quality as well as index scores for seven subscales: agreement, closeness, exposure to conflict, support, undermining, endorsement of partner's parenting, and division of labor. Overall positive coparenting (averaging items from agreement, closeness, support, endorsement of partner's parenting, and division of labor) and negative coparenting (averaging items from exposure to conflict and undermining) subscales were also generated (e.g., McDaniel et al., 2017).

In the current sample, the CRS subscales and global score demonstrated good to excellent internal consistency (see Table 3). As the division of labor subscale comprises two items, Spearman's rho (*ρ*) correlational analyses were conducted to assess internal consistency. The division of labor subscale demonstrated acceptable internal consistency in the female subsample (*ρ*(188) = 0.550, *p* < 0.001) and when assessing one member per coparenting dyad (*ρ*(214) = 0.535, *p* < 0.001). The internal consistency for the division of labor subscale was unacceptable (*ρ*(50) = 0.253, *p* = 0.075) in the male subsample, therefore the subscale was not used to test concurrent validity for male participants.

#### Coparenting children's emotions

The CCES, as outlined above, is designed to assess how coparents work together when responding to their children's emotions (see Table S1). Before responding to items, parents were instructed to focus on how they work with their child's other caregiver when responding to their child's emotions. The instructions included the statement: “emotions” are often referred to as “feelings” in daily life. All items were rated on a 5‐point scale (1 = *not at all true*; 5 = *very true*). Parents additionally indicated how frequently the exposure to conflict items occurred in a typical week when the parents and child were together (*never, sometimes [once or twice a week], often [once a day], very often [several times a day], not applicable*). The factor structure and psychometric properties of the CCES in this sample are reported in the “Results” section.

#### Interparental communication

The Communication Patterns Questionnaire‐Short Form (CPQ‐SF) was used to assess the concurrent validity between the CCES and a measure of interparental communication. The CPQ‐SF is an abbreviated version of the Communication Patterns Questionnaire (Christensen & Sullaway, 1984). The CPQ‐SF comprises 11 items that parents responded to on a nine‐point scale (1 = *very unlikely*, 9 = *very likely*). In the current study, the measure was scored using the subscale structure outlined by Futris et al. (2010). Futris et al. found the best model fit for the CPQ‐SF had 11 items separated into three subscales: criticize/defend, demand/withdraw, and positive interactions. In previous research, the subscales demonstrated good convergent validity with the Revised Dyadic Adjustment Scale and discriminated between participants with high and low martial adjustment (Futris et al., 2010). In the current study, criticize/defend and positive interactions demonstrated good to excellent internal consistency in the male subsample, female subsample, and independent subsample. The demand/withdraw subscale showed good internal consistency in the male sample, but acceptable internal consistency in the female sample and independent subsample (see Table 2).

**TABLE 2:** Descriptive statistics for the CRS, CPQ‐SF, PSS, and PRQC for females, males, and a sample of one member from each coparenting dyad (independent subsample).

	Female		Male		Independent subsample	
	*M* (*SD*)	*α*	*M* (*SD*)	*α*	*M* (*SD*)	*α*
CRS total	3.86 (0.92)	0.80	4.34 (0.60)	0.66	3.92 (0.92)	0.80
Positive	4.50 (1.21)	0.96	5.02 (0.95)	0.94	4.56 (1.23)	0.95
Negative	1.30 (0.77)	0.80	1.25 (0.84)	0.84	1.33 (0.80)	0.80
Agreement	4.75 (1.25)	0.86	5.15 (1.14)	0.88	4.77 (1.30)	0.87
Closeness	4.29 (1.56)	0.86	4.69 (1.24)	0.82	4.36 (1.56)	0.86
Support	4.26 (1.61)	0.88	4.51 (1.47)	0.91	4.23 (1.66)	0.89
Endorsing other's parenting	4.63 (1.32)	0.90	5.39 (0.86)	0.83	4.75 (1.32)	0.90
Undermining	0.81 (1.09)	0.82	0.85 (1.16)	0.84	0.88 (1.18)	0.84
Division of labor^a^	4.31 (1.71)	–	5.41 (0.96)	–	4.50 (1.65)	–
Exposure to conflict	1.89 (0.85)	0.85	1.72 (0.85)	0.87	1.85 (0.85)	0.84
CPQ‐SF						
Demand/withdraw	3.42 (1.53)	0.65	3.09 (1.55)	0.80	3.37 (1.51)	0.67
Criticize/defend	3.66 (1.95)	0.81	3.23 (2.06)	0.91	3.64 (1.97)	0.81
Positive interactions	6.80 (1.73)	0.82	7.16 (1.50)	0.80	6.78 (1.75)	0.81
PSS	40.96 (8.97)	0.85	39.04 (10.63)	0.88	40.91 (9.61)	0.87
PRQC total	5.86 (1.04)	0.87	6.04 (0.85)	0.80	4.23 (1.03)	0.88

^a^ Spearman's Rho correlation analyses conducted for the two‐item subscale. Internal consistency reported in the “Method” section.

#### Parenting stress

The Parental Stress Scale (PSS; Berry & Jones, 1995) was used to assess concurrent validity between the CCES and a measure of parenting stress. The PSS specifically assesses the level of stress caregivers experience in their parenting role. The self‐report measure comprises 18 items responded to on a 5‐point rating scale (1 = *strongly disagree*; 5 = *strongly agree*). The PSS captures the negative (i.e., opportunity costs) and positive (i.e., emotional rewards) aspects of parenting. The total PSS score was used in the current study, with higher scores indicating higher levels of parenting stress. In previous research, the PSS demonstrated good internal consistency (Berry & Jones, 1995) and concurrent validity with measures of family functioning and parental anxiety (Zelman & Ferro, 2018). In the current study, the PSS demonstrated good to excellent internal consistency (see Table 2).

#### Intimate/romantic relationship quality

The Perceived Relationship Quality Component Inventory (PRQC; Fletcher et al., 2000) was used to assess concurrent validity between the CCES and a measure of intimate relationship quality. The measure was only administered to participants who identified as being in an intimate/romantic relationship with their coparent. Participants rated their current partner and relationship by responding to items on a 7‐point scale (1 = *not at all*; 7 = *extremely*). The PRQC has a total of 18 items divided equally across six subscales (satisfaction, commitment, intimacy, trust, passion, and love). As per recommendations from Fletcher et al. (2000), a global relationship quality score was calculated by aggregating the first item from each subscale. Higher total scores indicate more positive evaluations of the intimate partner/relationship and greater relationship quality. In previous research, the PRCQ total demonstrated good internal consistency (Fletcher et al., 2000). In the current study, the PRQC total demonstrated good to excellent internal consistency (see Table 2).

## RESULTS

### Preliminary analyses

Data were initially examined for missing responses and normality. Once screening and missing data were imputed using expectation maximization, the total sample was separated into two subsamples to ensure the exploratory factor analysis and confirmatory factor analysis were performed on independent subsamples. To create the subsamples, participant responses were first ordered by date of survey completion to limit historical effects. As recruitment was open from January 2018 to December 2020, it was important to control for the potential effects of the COVID‐19 pandemic on family functioning, coparenting, and parenting stress. The participants were then allocated ID codes and the sample was split based on even and odd ID codes (i.e., alternate participants moved into separate subsamples to ensure an equal number of pre‐ and during‐ pandemic participants in each group). The subsamples were screened to ensure coparent dyads were separated to avoid dependent data (i.e., two participants reporting on the same coparenting relationship).

### Main analyses

#### Exploratory factor analysis

Exploratory factor analyses (EFA) were conducted to examine the factor structure of the CCES. Before performing the EFA, the suitability of the data was assessed. Correlation analyses were conducted to examine the strength of relationships between items. Based on the correlation matrix, items with weak (<0.2) or extremely strong correlations (>0.8) across multiple items were removed (Field, 2013). The Kaiser‐Meyer‐Olkin (KMO) measure of sampling adequacy and the Barlett's test of sphericity were also used to ensure the data were appropriate for EFA. The overall KMO index for the current data was 0.89 and was deemed meritorious (Kaiser, 1974). The Barlett's test of sphericity was statistically significant (*p* < 0.001) suggesting data were appropriate to include in the analysis. An EFA, using principal axis factoring with oblique (oblimin) rotation, was conducted and the factor structure was assessed. Oblique rotation was conducted as the factors were expected to correlate (Field, 2013). Items that cross‐loaded onto two or more factors at larger than 0.4 were removed, and the EFA was rerun.

The final EFA revealed a two‐factor solution: support/cooperation (eigenvalue 6.21) and undermining (eigenvalue 1.57). The scree plot confirmed two factors should be extracted. The support/cooperation factor comprised items 1, 3, 6, 12, 14, 19, 23, 37, 41 and explained 51.75% variance. The support/cooperation factor comprises a mixture of items modified from the original CRS subscales of coparenting closeness, coparenting support, endorsing partner parenting, coparent agreement, and division of labor. Higher scores on this subscale indicate that coparents are more supportive, work collaboratively, affirm each other's parenting abilities, and respect and acknowledge each other's contributions to responding to their children's emotions. The undermining factor comprised items 7, 13, and 18, which explained 13.12% variance. The undermining factor depicts parents' experiences of criticism and disparagement in the coparenting relationship. Parents with high scores on this subscale may feel that their ability to respond to their children's emotions is questioned and/or belittled by the other parent. Table 3 displays the final factor structure.

**TABLE 3:** Factor structure of the Coparenting Children's Emotion Scale.

Scale items	Factor 1	Factor 2
	Support/cooperation	Undermining
19. The other parent/caregiver is sensitive to our child's feelings	0.88	
3. I believe the other parent/caregiver is good at responding to our child's emotions	0.87	
41. The other parent/caregiver and I use similar strategies when helping our child with his/her emotions	0.78	
37. The other parent/caregiver does not like to be bothered by our child's intense emotions. (R)	0.78	
12. We often discuss the best way to meet our child's emotional needs	0.76	
23. When I'm overwhelmed by my child's emotions, the other parent/caregiver provides me with the extra support I need	0.73	
1. The other parent/caregiver asks my opinion about how we can best respond to our child's emotions	0.73	
14. The other parent/caregiver still wants to do his/her own thing instead of being available when our child needs emotional support. (R)	0.71	
6. The other parent/caregiver tells me I am doing a good job of helping our child with his/her emotions	0.40	
7. The other parent/caregiver criticizes the way I help our child with his/her emotions		0.82
18. The other parent/caregiver tries to show that he/she is better than me at responding to our child's emotions		0.72
13. The other parent/caregiver does not trust my ability to help our child when they are experiencing intense emotions		0.68

*Note*: (R) indicates the item was reverse scored.

#### Confirmatory factor analysis

The EFA factor structure was tested using data from the second subsample in a confirmatory factor analysis (CFA) using AMOS 26 Graphics. The goodness of model fit was assessed by reviewing the maximum likelihood chi‐square (*χ* ^2^), comparative fit index (*CFI*), normed fit index (*NFI*), Tucker–Lewis index (*TLI*), goodness‐of‐fit index (*GFI*), incremental fit (*IFI*), and root square error approximation (*RMSEA*). As the chi‐square test is highly sensitive to sample size, numerous additional indices were used. Good model fit is indicated by *CFI*, *NFI*, and *TLI* > 0.95, *GFI* and *IFI* > 0.90, and *RMSEA* < 0.06 (Dardas & Ahmad, 2014; Hu & Bentler, 1999; West et al., 2012). The modification indices guided model refinement (see Table 4).

**TABLE 4:** Model fit statistics from the confirmatory factor analysis for the Coparenting Children's Emotion Scale.

	Model fit indices						
	*χ* ^2^	*CFI*	*NFI*	*TLI*	*IFI*	*GFI*	*RMSEA*
EFA model	108.57 (*p* < 0.001)	0.94	0.88	0.92	0.94	0.89	0.088
Refined model	61.92 (*p* = 0.03)	0.98	0.92	0.97	0.98	0.92	0.057
Alternative model	76.35 (*p* = 0.001)	0.96	0.91	0.94	0.96	0.91	0.076

The CFA indicated that the EFA factor structure had acceptable fit. Modification indices suggested the relationship between item 37 and item 14 was impairing model fit. Based on the readability of the items, item 14 was removed (i.e., The other parent/caregiver still wants to do his/her own thing instead of being available when our child needs emotional support [R]). The refined model (EFA model without item 14) demonstrated excellent model fit across most indices. An alternative model was run to determine whether removing item 37 (i.e., The other parent/caregiver does not like to be bothered by our child's intense emotions [R]) and retaining item 14 would produce a better model fit. This alternative model (EFA model without item 37) fit was acceptable. The best model fit was the refined model, which comprised eight items on the support/cooperation subscale and all three items from the undermining subscale. See Table S2 for the final version of the scale.

#### Reliability: Internal consistency

Cronbach's alpha analyses were conducted to assess the internal consistency of the CCES in the current sample. Data were analyzed in three subgroups: (1) females, (2) males, and (3) a subsample containing one member from each coparent dyad to ensure data were independent (i.e., independent subsample). The support/cooperation subscale demonstrated excellent internal consistency in the female subsample and independent subsample, and good internal consistency in the male subsample (see Table 5). The undermining subscale also demonstrated good reliability in the male subsample and independent subsample, and acceptable internal consistency in the female subsample (see Table 5).

**TABLE 5:** Descriptive statistics and internal consistency of the CCES subscales for females, males, and a subsample of one member from each coparenting dyad.

	Female		Male		Independent subsample	
	*M* (*SD*)	*α*	*M* (*SD*)	*α*	*M* (*SD*)	*α*
Support/cooperation	3.85 (0.98)	0.91	4.40 (0.73)	0.85	3.89 (1.00)	0.91
Undermining	1.03 (0.56)	0.79	1.15 (0.54)	0.83	1.09 (0.59)	0.82

#### Convergent and concurrent validity

Spearman's Rho (*ρ*) correlation analyses were used to assess validity as the data for the CCES subscales were not normally distributed. Data were examined separately for females and males as previous research has indicated differences in their perceptions of the coparenting relationship (e.g., Feinberg et al., 2012). Validity was assessed by examining correlations between the CCES subscales and theoretically related constructs (i.e., overall coparenting, interparental communication, parenting stress, romantic relationship quality). Spearman's Rho correlation coefficients (*ρ*) are reported in Table 6. Effect sizes were categorized as: small (*ρ* ≥ 0.1), moderate (*ρ* ≥ 0.3), and strong/large (*ρ* ≥ 0.5; Cohen, 1988).

**TABLE 6:** Spearman's rho coefficients (*ρ*) for correlations between the CCES support/cooperation and CCES undermining subscales and the CRS, CPQ‐SF, PSS, and PRQC for females, males, and an independent subsample of one member from each coparenting dyad.

	CCES support/cooperation			CCES undermining		
	Female	Male	Independent subsample	Female	Male	Independent subsample
CRS: Total^a^	0.76***	0.70***	0.76***	−0.37***	−0.49***	−0.36***
Positive	0.82***	0.84***	0.82***	−0.48***	−0.54***	−0.50***
Negative	−0.54***	−0.60***	−0.54***	0.52***	0.68***	0.59***
Agreement	0.59***	0.73***	0.61***	−0.52***	−0.57***	−0.49***
Closeness	0.58***	0.71***	0.63***	−0.39***	−0.47***	−0.44***
Supportive	0.82***	0.84***	0.81***	−0.48***	−0.63***	−0.54***
Endorsing other's parenting	0.79***	0.43***	0.73***	−0.38***	−0.24	−0.30***
Undermining	−0.59***	−0.60***	−0.56***	0.59***	0.77***	0.63***
Division of labor	0.53***	0.57***	0.53***	−0.28***	−0.61***	−0.24***
Exposure to conflict	−0.24***	−0.42***	−0.22***	0.27***	0.36*	0.26***
CPQ‐SF^b^						
Demand/withdraw	−0.60***	−0.61***	−0.60***	0.42***	0.39***	0.42***
Criticize/defend	−0.42***	−0.51***	−0.41***	0.55***	0.49***	0.53***
Positive interactions	0.64***	0.81***	0.66***	−0.39***	−0.51***	−0.43***
PSS Total^c^	−0.18*	−0.38***	−0.23***	0.25***	0.55***	0.27***
PRQC Total^d^	0.47***	0.70***	0.48***	−0.29***	−0.59***	−0.39***

^a^ *n*_Females_ = 187, *n*_Males_ = 50, *n*_Independent subsample_ = 213.

^b^ *n*_Females_ = 187, *n*_Males_ = 50, *n*_Independent subsample_ = 209.

^c^ *n*_Females_ = 187, *n*_Males_ = 49, *n*_Independent subsamplee_ = 221.

^d^ *n*_Females_ = 161, *n*_Males_ = 42, *n*_Independent subscample_ = 173.

* *p* < 0.05.

*** *p* < 0.001.

##### Coparenting relationships

Moderate to strong correlations were expected between the CCES subscales and the CRS. As expected, Spearman's Rho analyses indicated a strong positive correlation between the CCES support/cooperation subscale and the CRS total, and a moderate negative correlation between CCES undermining subscale and total CRS.

When examining convergent validity at the subscale level, the CCES support/cooperation subscale was positively correlated with CRS agreement, closeness, support, endorsing, and division of labor, and positive coparenting subscale. The effect size of these correlations was moderate to strong. The CCES support/cooperation subscale was also negatively correlated with CRS undermining, exposure to conflict, and negative coparenting subscales. As expected, the CCES undermining subscale was strongly correlated with CRS undermining, and moderately correlated with CRS exposure to conflict. Additionally, the CCES undermining subscale was negatively correlated with CRS agreement, closeness, support, endorsing, division of labor, and positive coparenting subscales, with effect sizes ranging from moderate to large.

##### Interparental Communication

Moderate correlations were expected between the CCSE subscales and CPQ‐SF subscales, as interparental communication plays a central role in the coparenting relationship. Spearman's rho analyses revealed good concurrent validity as both CCES subscales were significantly associated with communication patterns. Specifically, the CCES support/cooperation coparenting subscale was negatively correlated with CPQ‐SF demand/withdraw and criticize/defend communication styles, and positively correlated with positive interactions. The CCES undermining subscale was positively correlated with CPQ‐SF demand/withdraw and criticize/defend communication styles, and negatively correlated with CPQ‐SF positive interactions. All correlations were moderate to large in strength.

##### Parental stress

Small to moderate correlations were expected between the CCES subscales and PSS, as research indicates the coparenting relationship influences parents' experience of childrearing stress (Feinberg & Sakuma, 2011). Spearman's rho analyses revealed small to moderate negative correlations between CCES support/cooperation subscale and the PSS. Furthermore, females reported a small positive correlation between CCES undermining subscale and PSS, whereas males reported a strong positive correlation. A Fisher's *z* test for equality of correlation was conducted to assess whether correlations between CCES undermining and PSS differed across males and females. The results indicated the correlation magnitudes were significantly different (*z* = 2.30, *p* = 0.011).

##### Romantic relationship quality

To demonstrate concurrent validity, small to moderate correlations were expected between CCES subscales and the PRQC, as the coparenting relationship is related yet distinct from the romantic relationship (e.g., Maršanić & Kušmić, 2013). Spearman's Rho analyses showed a moderate to strong positive correlation between overall romantic relationship quality (PRQC total) and the CCES support/cooperation subscale and a negative correlation with the CCES undermining subscale. A Fisher's *z* test for equality of correlation indicated the magnitude of these associations differed significantly between males and females (CCES support/PRQC: *z* = 1.936, *p* = 0.026; CCES undermining/PRQC: *z* = 2.145, *p* = 0.016).

## DISCUSSION

This study outlined the development, factor structure, reliability, and validity of the CCES, a new measure designed to assess coparenting children's emotions. The CCES was developed in response to gaps in coparenting and emotion socialization literature, and the need for a targeted, emotion‐focused coparenting measure. The CCES specifically assesses how supportive, cooperative, and undermining coparents are when working together to respond to their children's emotions. Overall, the results indicate that CCES is a psychometrically sound measure of coparenting children's emotion as it demonstrated good internal consistency and validity.

The CCES captures the supportiveness/cooperation and undermining that occurs between coparents when responding to their children's emotions. According to the coparenting literature, parents' supportive and undermining efforts provide substantial insight into the nature and functioning of coparenting relationships (e.g., Belsky et al., 1996; Mangelsdorf et al., 2011). The CCES support/cooperation subscale reflects a well‐established construct within the coparenting literature, which describes how coparents value and respect each other's parenting, and work collaboratively and cooperatively together (e.g., Feinberg, 2003; Margolin et al., 2001; McHale, 1995; Van Egeren & Hawkins, 2004). The CCES undermining subscale reflects the extent to which one or both coparents belittle, criticize, and disparage each other (Belsky et al., 1996; Feinberg, 2003; Van Egeren & Hawkins, 2004). These CCES subscales allow researchers to assess two influential coparenting dynamics within the context of emotion socialization. For example, coparenting interventions (e.g., Family Foundations; Feinberg & Kan, 2008) typically focus on addressing supportive and undermining coparenting as these dynamics have profound impact on parenting (Bonds & Gondoli, 2007; Margolin et al., 2001; Morrill et al., 2010) and child outcomes (e.g., internalizing and externalizing difficulties, physical health; Teubert & Pinquart, 2010, Zemp et al., 2020). The support/cooperation and undermining dimensions of coparenting are prominently and consistently examined throughout the literature (e.g., Belsky et al., 1996; Feinberg, 2003; Margolin et al., 2001; McHale, 1995; McHale et al., 2019; McHale & Irace, 2011).

The CCES subscales demonstrated good internal consistency, convergent validity, and concurrent validity. As expected, the CCES support/cooperation and undermining subscales correlated with theoretically related constructs (i.e., global coparenting relationship quality, interparental communication, stress, intimate/romantic relationship quality). The moderate to strong correlations between the CCES subscales and the CRS total and subscales indicate good convergent validity as the measures assess similar, yet distinct, aspects of the coparenting relationship. It is important that the CCES, a domain‐specific measure, provides additional information about coparenting in the context of emotion socialization compared a global measure of coparenting relationship quality. Future research should examine whether the CCES uniquely predicts parent emotion socialization practices above and beyond the CRS.

To date, extant research has not specifically examined how coparenting children's emotions relates to interparental communication patterns, parenting stress, or relationship quality; therefore, findings from previous research on global coparenting support and undermining informed expectations for concurrent validity of the CCES subscales. Current findings indicate that the CCES subscales were moderately to strongly correlated with interparental communication patterns, which is consistent with coparenting literature (e.g., Shimkowski & Schrodt, 2012). For example, research by Zemp et al. (2020) found a moderate correlation between coparenting and constructive interparental conflict. The correlations between CCES subscales and interparental communication patterns subscales are also consistent with previous research on the CRS, which showed strong correlations between the CRS total score and ineffective arguing and couple conflict (Feinberg et al., 2012). Furthermore, the small to moderate correlations between CCES subscales and parental stress are consistent with previous research, which demonstrated small to moderate correlations between supportive and undermining coparenting and parental stress in mothers and fathers (e.g., Solmeyer & Feinberg, 2011).

The moderate correlations between CCES and PRQC align with theory and empirical research that suggest the coparenting relationship and intimate/romantic relationship are related yet distinct subsystems (Maršanić & Kušmić, 2013). Previous research showed coparenting relationship quality (as measured by the CRS) was strongly correlated with couple love (i.e., the extent to which their relationship is loving, giving, committed, intimate, and cohesive; Feinberg et al., 2012). Similarly, in a sample of parents of children aged 3 years old, Le et al. (2016) found strong negative correlations between romantic relationship quality and CRS undermining, and strong positive correlations between romantic relationship quality and CRS support.

All correlations for males, females, and the independent subsample were in the expected direction; however, males, compared to females, reported stronger correlations between both CCES subscales and romantic relationship quality, and CCES undermining and parental stress. Previous research indicates that females and males report differences in their perceptions of the coparenting relationship (Feinberg et al., 2012), as such, these results are unsurprising. The father vulnerability hypothesis offers a potential explanation for the gender differences between male and female caregivers (Belsky et al., 1984; Cummings et al., 2004). Researchers posit male caregivers experience increased vulnerability because social conventions regarding the parenting roles of father are less distinct and scripted compared to the roles of mothers (Cummings et al., 2004; Davies et al., 2009; Parke, 2002). As a result, fathers typically have less distinct boundaries between their relationships and are less able to compartmentalize between the romantic relationship and coparenting relationship, which can result in spill‐over effects (i.e., negative and/or positive affect transferring between family subsystems; Katz & Gottman, 1996; Pedro et al., 2012). In comparison, mothers may be better at establishing emotional boundaries between their family subsystems (e.g., romantic relationship, coparenting relationship; Belsky et al., 1991). Furthermore, fathers may be more susceptible to parenting stress in the face of coparent undermining (Parke, 2002; Peltz et al., 2018). Research suggests that fathers' perceptions of their parenting ability is influenced by mothers' attitudes about paternal competence and involvement, whereas mothers do not seem as effected by their coparents' opinions (Van Egeren & Hawkins, 2004). It is also possible that undermining in the coparenting relationship and fathers' stress are compounded by maternal gatekeeping, a coparenting process in which female caregivers may hinder or control male caregivers' involvement with their children (Stevenson et al., 2014). Although the father vulnerability hypothesis offers a potential explanation, empirical findings are mixed, with evidence both supporting (e.g., Davies et al., 2009; Stevenson et al., 2014) and refuting (e.g., Ponnet et al., 2013) the theory. Despite gender differences, the direction and magnitude of correlations between CCSE subscales and romantic relationship quality and CCES undermining and parental stress provide evidence of good concurrent validity.

### Limitations and future directions

The current study has several limitations. Firstly, the study was reliant on self‐report measures, which are inherently subjective. To improve confidence in the criterion validity of the CCES, participants' scores could be compared against observational measures of coparenting (e.g., McHale et al., 2001). For example, future research could explore how self‐reports of coparenting children's emotions relate to observations of coparenting dynamics when children engage in tasks that elicit emotion (e.g., frustration during a complex puzzle box task; Eisenberg et al., 1997). Secondly, correlational analyses using the CPQ‐SF demand/withdraw subscale should be interpreted with caution as the subscale showed low internal consistency in the female subsample and independent subsample. Additionally, the characteristics of the sample may limit generalisability. Despite efforts to be inclusive of all family structures, the sample was predominantly female and composed of married/de‐facto, co‐habiting coparents. Furthermore, most participants were raising one to two children who, on average, were primary school aged. Given this, it is unclear whether the CCES would display similar validity and reliability when used with diverse coparent dyads (e.g., coparents who are divorced/separated or family members) and parents of toddlers and adolescents. It is also important to test the CCES with coparents in high conflict relationships to determine whether the same factor structure and psychometric properties are demonstrated in different samples. Predictive validity is another psychometric property that still needs to be established. Future research with the CCES could examine predictive validity by using measures of child internalizing and externalizing difficulties (e.g., Child Behavior Checklist—Achenbach & Rescorla, 2001; Strengths and Difficulties Questionnaire—Goodman, 2001). Additionally, further research may continue refining the CCES. New items that focus on the survey respondent's behaviors towards their coparent could be trailed (e.g., “when the other parent is overwhelmed by our child's emotions, I provide the extra support he/she needs”). Furthermore, “your child's other parent” may be used in items instead of “the other parent”.

In future research, the CCES can be used in conjunction with measures of emotion socialization practices to further assess construct validity and provide insight into the complexity of family emotional socialization. For example, researchers may examine whether undermining in coparenting children's emotion is positively correlated with emotion unavailability and emotion disapproving practices (e.g., criticizing children's emotions), and whether support/cooperation in emotion coparenting is positive correlated with emotional sensitivity and emotion coaching (e.g., noticing and validating children's emotions). Gaining more information about the role of coparenting children's emotions will assist researchers and practitioners to develop and refine emotion‐focused parenting interventions. Additionally, researchers and practitioners who assess emotion‐focused parenting programs may choose to use the CCES instead of the CRS as it is a specific measure of how parents are working together when using emotion socialization practices.

### Implications and conclusion

This study outlined the development and psychometric assessment of a new measure of coparenting children's emotions. The CCES fills a gap in coparenting and emotion socialization literature, by providing a domain‐specific measure to assess how supportive, cooperative, and undermining coparents are when working together to respond to their children's emotion. Overall, the CCES demonstrated good internal consistency and validity within the current sample, which provides preliminary support for the measure. Future research could assess the psychometric properties of the measure in diverse samples.

The CCES provides researchers and practitioners with a targeted tool that can help expand emotion socialization and coparenting research and develop and evaluate emotion‐focused parenting programs. Given the interdependency within family systems, a greater understanding of coparenting children's emotions will provide insights into the emotion socialization processes that occur within the family unit. For example, the CCES can be used to examine coparenting in an emotional context, such as how coparenting children's emotions contributes to emotion socialization by influencing parent emotion socialization practices and children's emotional competence. The CCES can also be used to evaluate coparenting children's emotions as an outcome or moderator in emotion‐focused parenting programs. By understanding the moderating effects of supportiveness/cooperation and undermining in coparenting children's emotions, researchers and practitioners can develop and refine emotion‐focused parenting programs and make recommendations about which families will benefit most from attending. It is hoped that researchers and practitioners will use the CCES to explore the ways parents work together in responding to their children's emotions.

## Supporting information


**Table S1.**.

**Table S2.**.

## Footnotes

^1^
Parents/caregivers raising a child (aged 1–18 years old) with another adult were eligible to participate. Parents were able to participate regardless of their relationship with the other parent/caregivers (e.g., married, divorced/separated, family, and friends), and were not required to be the child's biological parent nor live with the child.
