# Supplementary material for: Development and psychometric evaluation of a new domain‐specific coparenting measure: Coparenting Children's Emotion Scale
Source: Fam Process. 2024 Aug 21;63(4):2581–602. doi: 10.1111/famp.13031 (PMC11659102; doi:10.1111/famp.13031)
Supplement: Supplementary file 1 — Table S1.. [file FAMP-63-2581-s001.pdf]

Table S1

Items Developed for the Coparenting Children's Emotion Scale

CCES item	Original items
1. The other parent/caregiver asks my opinion about how we can best respond to our child's emotions.	3. My partner asks my opinion on issues related to parenting[a]
2. It is easier to talk to my child about his/her feelings when the other parent is not present.	8. It is easier and more fun to play with the child alone than it is when my partner is present too[a]
3. I believe the other parent is good at responding to our child's emotions.	1. I believe my partner is a good parent[a]
4. The other parent and I have the same goals for our child's emotional wellbeing.	6. My partner and I have the same goals for our child[a]
5. The other parent likes to spend time with our child when the child is happy but expects me to respond to our child when he/she is sad. (R)	5. My partner likes to play with our child and then leave dirty work to me[a] (R)
6. The other parent tells me I am doing a good job of helping our child with his/her emotions.	10. My partner tells me I am doing a good job or otherwise lets me know I am being a good parent[a]
7. The other parent criticises the way I help our child with his/her emotions.	12. My partner sometimes makes jokes or sarcastic comments about the way I am as a parent[a]
8. The other parent pays a great deal of attention to our child's emotion.	4. My partner pays a great deal of attention to our child[a]
9. The other parent and I have different ideas about how to respond to our child's sadness.	9. My partner and I have different ideas about how to raise our child[a] (R) and 11. My partner and I have different ideas regarding our child's eating, sleeping, and other routines[a] (R)
10. The other parent likes to spend time with our child when the child is happy but expects me to respond to our child when he/she is angry. (R)	5. My partner likes to play with our child and then leave dirty work to me[a] (R)
11. The other parent/caregiver and I have arguments about how to respond to our child's emotions, when the child is not present	Created by CA
12. We often discuss the best way to meet our child's emotional needs.	19.We often discuss the best way to meet our child's needs[a]

CCES item	Original items
13. The other parent does not trust my ability to help our child when they are experiencing intense emotions.	13. My partner does not trust my abilities as a parent[a]
14. The other parent still wants to do his/her own thing instead of being available when our child needs emotional support. (R)	7. My partner still wants to do his or her own thing instead of being a responsible parent[a] (R)
15. The other parent does not like to be bothered by our child’s sadness.	29. My partner doesn't like to be bothered by our child[a] (R)
16. The other parent and I have different ideas about how to respond to our child’s anger.	9. My partner and I have different ideas about how to raise our child[a] (R) and 11. My partner and I have different ideas regarding our child's eating, sleeping, and other routines[a] (R)
17. The other parent appreciates how hard I work to support our child’s emotional wellbeing.	25. My partner appreciates how hard I work at being a good parent[a]
18. The other parent tries to show that he/she is better than me at responding to our child’s emotions.	16. My partner tries to show that she or he is better than me at caring for our child[a]
19. The other parent is sensitive to our child's feelings.	14. My partner is sensitive to our child's feelings and needs[a]
20. The other parent does not talk to our child about emotions as often as I think he/she should. (R)	20. My partner does not carry his or her fair share of the parenting work[a] (R)
21. The other parent and I have different standards for how our child should behave when they are worried. (R)	15. My partner and I have different standards for our child’s behaviour[a] (R)
22. The other parent uses strategies that I have asked him/her not to use when responding to our child’s emotions	11. My partner uses parenting techniques that I have asked him/her not to use[b]
23. When I’m overwhelmed by my child’s emotions, the other parent provides me with the extra support I need.	26. When I'm at my wits end as a parent, partner gives me extra support I need[a]
24. The other parent has a lot of patience when responding to our child’s emotions.	18. My partner has a lot of patience with our child[a]

**CCES item**	**Original items**
25. The other partner and I have different ideas about how to respond to our child's worry.	9. My partner and I have different ideas about how to raise our child[a] (R) and 11. My partner and I have different ideas regarding our child's eating, sleeping, and other routines[a] (R)
26. In general, I think we work well together to support our child's emotional wellbeing.	12. In general, I feel we work well together with our child[b]
27. The other parent does not like to be bothered by our child's anger.	29. My partner doesn't like to be bothered by our child[a] (R)
28. The other parent and I have different ideas about how to respond to our child's happiness.	9. My partner and I have different ideas about how to raise our child[a] (R) and 11. My partner and I have different ideas regarding our child's eating, sleeping, and other routines[a] (R)
29. The other parent and I have different ideas about how to respond to our child's emotion.	9. My partner and I have different ideas about how to raise our child[a] (R) and 11. My partner and I have different ideas regarding our child's eating, sleeping, and other routines[a] (R)
30. The other parent makes me feel like I am providing our child with the best emotional support possible.	27. My partner makes me feel like I'm the best possible parent for our child[a]
31. The other parent is willing to make personal sacrifices to prioritise our child's emotions.	23. My partner is willing to make personal sacrifices to help take care of our child[a]
32. The other parent and I have different standards for how our child should behave when they are sad. (R)	15. My partner and I have different standards for our child's behaviour[a] (R)
33. The other parent ignores me when I ask his/her to help me respond to our child's emotions	4. When I ask for my partner's help when trying to get our child to bed, he/she ignores me[b]
34. The other parent does not like to be bothered by our child's worry.	29. My partner doesn't like to be bothered by our child[a] (R)
35. The other parent and I have different standards for how our child should behave when they are angry. (R)	15. My partner and I have different standards for our child's behaviour[a] (R)
36. The other parent and I have arguments about the best way to respond to our child's emotions. (R)	Created by CA

CCES item	Original items
37. The other parent/caregiver does not like to be bothered by our child's strong/intense emotions.	29. My partner doesn't like to be bothered by our child[a] (R)
38. The other parent supports me when I am responding to our child's emotions.	Created by CA.
39. The other parent and I use similar strategies when helping our child with his/her emotions	7. My partner and I use similar parenting techniques[a]
40. The other parent and I calmly discuss how we both want to help our child manage his/her emotions.	Created by CA
41. The other parent likes to spend time with our child when the child is happy but expects me to respond to our child when he/she is worried. (R)	5. My partner likes to play with our child and then leave dirty work to me[a] (R)
42. The other parent and I have arguments about how to respond to our child's emotions, when the child is present.	How often in a typical week, when all 3 of you are together, do you: 32. Argue with your partner about your child, in the child's presence? [a]
43. The other parent disregards my feelings in front of our child.	Created by CA
44. The other parent supports me in front of our child, even though he/she may not agree with how I am responding to our child's emotions (R)	14. When my partner doesn't agree with how I am handling our child, he/ she still backs me up in front of our child[b]
45. The other parent criticises how I manage our child's emotions, when our child is present.	5. My partner criticizes my parenting in front of our child[b]
46. I make negative comments to my child about how the other parent responds to his/her emotions (e.g., I think Dad can be very nasty to you when you're sad).	Created by CA

Notes.

[a]Items from Feinberg et al. (2012)

[b]Items adapted from Stright and Bales (2003)
