# Supplementary material for: Development and psychometric evaluation of a new domain‐specific coparenting measure: Coparenting Children's Emotion Scale
Source: Fam Process. 2024 Aug 21;63(4):2581–602. doi: 10.1111/famp.13031 (PMC11659102; doi:10.1111/famp.13031)
Supplement: Supplementary file 2 — Table S2.. [file FAMP-63-2581-s002.pdf]

Table S2

Finalised Items and Subscale Structure of the Coparenting Children's Emotion Scale

Subscales	Items
**Support/cooperation**	The other parent/caregiver is sensitive to our child's feelings.
	I believe the other parent/caregiver is good at responding to our child's emotions.
	The other parent/caregiver and I use similar strategies when helping our child with his/her emotions
	The other parent/caregiver does not like to be bothered by our child's intense emotions (R)
	We often discuss the best way to meet our child's emotional needs.
	When I'm overwhelmed by my child's emotions, the other parent/caregiver provides me with the extra support I need.
	The other parent/caregiver asks my opinion about how we can best respond to our child's emotions.
	The other parent/caregiver tells me I am doing a good job of helping our child with his/her emotions.
**Undermining**	The other parent/caregiver criticises the way I help our child with his/her emotions.
	The other parent/caregiver tries to show that he/she is better than me at responding to our child's emotions.
	The other parent/caregiver does not trust my ability to help our child when they are experiencing intense emotions.
